# FLASH-TB: an Application of Next-Generation CRISPR to Detect Drug Resistant Tuberculosis from Direct Sputum

**DOI:** 10.1128/jcm.01634-22

**Published:** 2023-04-03

**Authors:** Trinh Thi Bich Tram, Vu Thi Ngoc Ha, Le Pham Tien Trieu, Philip M. Ashton, Emily D. Crawford, Do Dang Anh Thu, Nguyen Le Quang, Guy E. Thwaites, Timothy M. Walker, Catherine Anscombe, Nguyen Thuy Thuong Thuong

**Affiliations:** a Oxford University Clinical Research Unit, Ho Chi Minh City, Vietnam; b Centre for Tropical Medicine and Global Health, Nuffield Department of Medicine, University of Oxford, Oxford, United Kingdom; c Institute of Infection, Veterinary and Ecological Sciences, University of Liverpool, Liverpool, United Kingdom; d Department of Microbiology and Immunology, University of California San Francisco, San Francisco, California, USA; University of Manitoba

**Keywords:** CRISPR, FLASH, *Mycobacterium tuberculosis*, RNA guide, drug resistance, sequencing

## Abstract

Offering patients with tuberculosis (TB) an optimal and timely treatment regimen depends on the rapid detection of Mycobacterium tuberculosis (Mtb) drug resistance from clinical samples. Finding Low Abundance Sequences by Hybridization (FLASH) is a technique that harnesses the efficiency, specificity, and flexibility of the Cas9 enzyme to enrich targeted sequences. Here, we used FLASH to amplify 52 candidate genes probably associated with resistance to first- and second-line drugs in the Mtb reference strain (H37Rv), then detect drug resistance mutations in cultured Mtb isolates, and in sputum samples. 92% of H37Rv reads mapped to Mtb targets, with 97.8% of target regions covered at a depth ≥ 10X. Among cultured isolates, FLASH-TB detected the same 17 drug resistance mutations as whole genome sequencing (WGS) did, but with much greater depth. Among the 16 sputum samples, FLASH-TB increased recovery of Mtb DNA compared with WGS (from 1.4% [IQR 0.5-7.5] to 33% [IQR 4.6-66.3]) and average depth reads of targets (from 6.3 [IQR 3.8-10.5] to 1991 [IQR 254.4-3623.7]). FLASH-TB identified Mtb complex in all 16 samples based on IS*1081* and IS*6110* copies. Drug resistance predictions for 15/16 (93.7%) clinical samples were highly concordant with phenotypic DST for isoniazid, rifampicin, amikacin, and kanamycin [15/15 (100%)], ethambutol [12/15 (80%)] and moxifloxacin [14/15 (93.3%)]. These results highlighted the potential of FLASH-TB for detecting Mtb drug resistance from sputum samples.

## INTRODUCTION

Tuberculosis (TB), caused by Mycobacterium tuberculosis (*Mtb*) is one of the leading causes of death worldwide. Drug-resistant TB (DR-TB) is harder to treat and outcomes are worse (1). The COVID-19 pandemic reduced access to TB diagnosis and treatment, which has had devastating effects on global TB control. A total of 166,991 cases of drug rifampicin resistant (RR) TB were detected in 2021, a 17.0% fall since 2019 (2). The number of people with RR-TB who were enrolled on treatment in 2021 was also 11.0% lower than in 2019 (2). Importantly, these figures are suggestive of missed diagnoses, not of a decline in actual incidence, meaning tuberculosis diagnosis and treatment need to be made a priority.

World Health Organization (WHO) guidelines for the treatment of RR-TB are changing rapidly, with the latest iteration recommending a 6 month regimen that includes bedaquiline, pretomanid, and linezolid, with or without moxifloxacin (3). Along with this, the definition of pre-extensively drug resistance (pre-XDR) and extensively drug resistant TB (XDR-TB) have been refined, such that MDR-TB plus resistance to any fluoroquinolone is now ‘pre-XDR’, with the definition of XDR being now met with the addition of resistance to at least one of bedaquiline and linezolid (4). These changes highlight the rapid emergence of resistance to drugs in the new regimen (5). Advances in treatment recommendations are currently outpacing diagnostic capacity, which is currently woefully inadequate for these drugs. To achieve the goal of eliminating TB by 2035, rapid molecular diagnostic methods that are able to detect rifampicin resistance as well as resistance to the new and repurposed drugs are urgently needed.

Drug susceptibility testing (DST) is recommended by WHO in all cases (6, 7). Phenotypic DST (pDST) is infrastructure dependent, costly, and slow. While rapid molecular assays like GeneXpert (MTB/RIF and MTB/XDR) and line-probe assays (MTBDRplus and MTBDRsl) for first and second-line antibiotics reduce turnaround time by bypassing culture, these are limited by the number of drugs or mutations they can analyze. Whole genome sequencing (WGS) has already replaced phenotypic testing for first-line drugs in some high-income settings (8), but practice still depends on culture such that turnaround times remain slow. Performing WGS directly from clinical samples would improve the turnaround time but variant calling is less reliable due to low *Mtb* bacterial loads and high levels of contamination from human and other bacterial DNA (9). Early results for Deeplex Myc-TB, a commercial targeted next-generation sequencing assay, have been promising with a susceptibility prediction made for approximately 70% of samples graded negative, 1+, or 2+ by microscopy (10, 11). More data are still needed. The clustered regularly interspaced short palindromic repeats/CRISPR-associated protein 9 (CRISPR/Cas9) system is known for its efficiency, specificity, and programmability in gene editing (12). The Finding Low Abundance Sequences by Hybridization (FLASH) technique uses recombinant CRISPR/Cas9 coupled with multiplexed sets of guide RNAs to cleave sequences of interest into fragments appropriately sized for Illumina sequencing (13). This technology has been successfully applied to detect DR in respiratory fluid from pneumonia patients with Staphylococcus aureus, and from dried blood spots from malaria patients infected with Plasmodium falciparum. This targeted approach is rapid, inexpensive, and has a high multiplexing capacity, and is, thus, a promising technique for rapid diagnosis of DR from clinical samples in the future.

Here, we adopt the FLASH technique using guide RNAs for *Mtb* designed in an open source software tool (FLASHit) to detect DR-TB. We evaluate this FLASH-TB method using the laboratory H37Rv *Mtb* reference strain, cultured isolates, and sputum samples from patients with TB.

## MATERIALS AND METHODS

### Ethics statement.

Written informed consent was obtained from all participants prior to sample collection. Protocols were approved by the Institutional Review Boards of Pham Ngoc Thach Hosptial, Ho Chi Minh City, Vietnam and the Oxford Tropical Research Ethics Committee, UK.

### Samples.

Firstly, the H37Rv *Mtb* laboratory strain was cultured on Löwenstein-Jensen media and used to assess the limit of detection of FLASH-TB after serial dilution. Secondly, 4 cultured clinical isolates from pulmonary TB patients were selected from an existing collection for which DNA had previously been extracted. Those isolates with the most DR mutations detected by Mykrobe (14) and for which accompanying pDST also indicated resistance were selected. A fifth isolate that was drug-susceptible by Mykrobe and pDST was also selected. Thirdly, 16 sputum samples with at least 5 mL volume were selected consecutively to represent Ziehl-Neelsen smear microscopy scores of 1+ (6 samples), 2+ (6 samples) and 3+ (4 samples). These were as a part of an ongoing population-based study of tuberculosis in Ho Chi Minh City, Viet Nam. Samples were collected before the start of anti-TB therapy, then decontaminated by N-acetyl-L-cysteine and 2% NaOH. Routine tests were carried out for TB confirmation, including Ziehl-Neelsen smear, GeneXpert MTB/RIF, and MGIT culture. The remaining decontaminated samples were divided into 2 vials and kept at −20°C for further DNA extraction and experiment, respectively.

### *Mtb* DNA extraction.

Frozen sputum samples were thawed and sonicated for 20 min at 35 kHz and then heat inactivated in a thermal block for 30 min at 80°C, while *Mtb* cultures were inactivated for 2 h owing to their heavy bacterial load. Samples were stored at −20°C until DNA was extracted.

DNA from *Mtb* culture and sputum was extracted using a previously described mechanical disruption method (9, 15). Briefly, samples were incubated with 4M guanidine thiocyanate (GTC) lysis buffer (Sigma) to denature membrane protein of eukaryotic and Gram-negative cells, then treated with 40 μL DNase I solution including 35 μL buffer and 5 μL DNase I (Qiagen) at 37°C for 15 min to digest the released DNA from eukaryotic and Gram-negative cells. Samples were centrifuged at 13000g for 30 s and incubated at 95°C for 15 min to inactivate DNase I. After being washed and resuspended in 100 μL water, samples were then subjected to 3 rounds of bead-beating at 6m/s for 40 s. The beads were pelleted by centrifugation at 13000g for 10 min, and 50 μL of supernatant was cleaned with 90 μL of AMPure beads (Beckman Coulter). Samples were eluted in 25 μL water and quantified with a Qubit fluorimeter (Thermo Fisher Scientific).

### Design of CRISPR RNAs.

CRISPR RNA (crRNAs) were designed to target 52 genes previously used to identify *Mtb* (IS*6110* and IS*1081*) and predict DR (Tables S1 and 2), using a flexible computational tool called FLASHit (freely available at github.com/czbiohub/flash) as reported elsewhere (13). Briefly, we first determined a set of targetable 20-mer Cas9 sites, applying exclusion criteria: no homopolymers of length greater than 5, no runs of 2-base repeats greater than 3, no internal hairpins, GC content between 25% and 75%, any matches to a human sequence and E. coli (to avoid contaminated sequences from DNA of BL21, which was used to produce Cas-9 enzyme). Then, the tool defined an optimized guide set from this set, which satisfied the objective of maximizing the number of inter-guide inserts of optimal Illumina sequencing length (200 to 300 bp) covering each gene while minimizing the number of crRNAs. Consequently, a set of 499 RNA guides was used to target the 52 genes.

### Dual-guide RNAs preparation.

Dual-guide RNAs were prepared as described previously (13). Briefly, DNA oligonucleotide templates for crRNA and trans-activating CRISPR RNA (tracrRNA) were purchased from Integrated DNA Technologies (IDT). The crRNA template sequences were as follows: 5′-TAATACGACTCACTATAGNNNNNNNNNNNNNNNNNNNNGTTTTAGAGCTATGCTGTTTTG-3′, where the 20 Ns represent the 20 nucleotide target regions (Table S2). The tracrRNA template sequence was as follows: 5′-TAATACGACTCACTATAGGACAGCATAGCAAGTTAAAATAAGGCTAGTCCGTTATCAACTTGAAAAAGTGGCACCGAGTCGGTGCTTTTT-3′. Then, crRNA templates for every target were pooled. The tracrRNA and pooled crRNA templates were then transcribed using T7 RNA polymerase (NEB) for 2 h at 37°C. crRNAs and tracrRNAs were purified using AMPure magnetic beads and then annealed together at an equimolar ratio to form 40 μM dual-guide RNAs. These dual-guide RNAs were stored at single-use aliquots at −80°C for a year. Immediately prior to use, the dual-guide RNAs were annealed at 95°C for 30 s, then allowed to cool at room temperature.

### Preparation of FLASH-TB and WGS library.

The FLASH-TB library was prepared as described previously using a method of enzyme-based DNA library preparation (13). CRISPR/Cas9 protein were purified from soluble fraction of culture of BL-21 carrying Cas9 vector. The protein were concentrated and stored at −80°C in 50% glycerol for a year (13). Briefly, 5′ phosphate groups of the 5 ng DNA from cultured isolates or 100 ng DNA of each sputum sample were enzymatically cleaved using rAPid alkaline phosphatase (Sigma), which was subsequently deactivated with sodium orthovanadate (Sigma). The dephosphorylated DNA was added to a master mix containing a complex of CRISPR/Cas9 and dual-guide RNAs at 37°C for 2 h. The enzyme was deactivated with proteinase K (NEB) and removed with AMPure magnetic beads. Samples were dA-tailed using the NEBNext dA-Tailing Module and adapter-ligated using the NEBNext Ultra II reagents (NEB). Adapter dimer was removed by AMPure bead purifications and then products were indexed with 22 cycles of PCR using Q5 polymerase (NEB) and dual unique TruSeq i5/i7 barcode primers (Illumina). Following an AMPure bead purification, individual libraries were analyzed and quantified using KAPA Real-time Library Amplification Kits (Roche). Individual libraries were pooled based on the concentration of fragments of 250 to 600 bp.

WGS library for DNA from culture isolates or direct sputum were prepared using the Nextera XT kit (Illumina). All libraries were sequenced in 2 × 300bp Illumina MiSeq runs sequencing using MiseqV2 reagent kits (Illumina), multiplexing 6 samples per run for all FLASH libraries, or WGS libraries from direct sputum, or 18 for WGS from cultures.

### Sequencing data analysis.

FASTQ data generated on the Illumina MiSeq machine were trimmed using *bbduk*, mapped against the H37Rv reference genome (NC_000962.3) using *bwa mem* (16), and SNPs were called using GATK (version 3.8–1–0-gf15c1c3ef) in unified genotyper mode (17). These steps were executed by PHEnix pipeline (https://github.com/phe-bioinformatics/PHEnix). For sputum samples, the Clockwork pipeline (available at https://github.com/iqbal-lab-org/clockwork) was used to remove reads from non-tuberculosis mycobacteria, other bacteria, viruses, and humans. Only *Mtb* reads were kept for further analysis.

Custom python scripts were composed for tabulating the reads aligning to 50 DR genes to calculate the average target coverage and depth. Target coverage of 50 genes was determined as percentage of positions across gene sequence having depth greater than 10. *Mtb* complex were identified from WGS using Mykrobe predictor software v10.0.1 (https://github.com/Mykrobe-tools/mykrobe), while for FLASH-TB it was based on the presence of at least 30 reads for each of IS*6110* or IS*1081* elements, which are well established markers for the *Mtb* complex (18). Antibiotic resistance to isoniazid, rifampin, ethambutol, pyrazinamide, streptomycin, amikacin, and moxifloxacin was predicted with Mykrobe predictor software v10.0.1. For FLASH-TB and WGS, no resistance predictions were made for samples whose average target coverage was less than 80.0% and average target depth fell below 4. Graphs were generated by GraphPad Prism v7.03 (GraphPad Software) or R program v4.0.2 (19).

## RESULTS

### Performance of FLASH-TB applied to H37Rv DNA.

**FIG 1:**
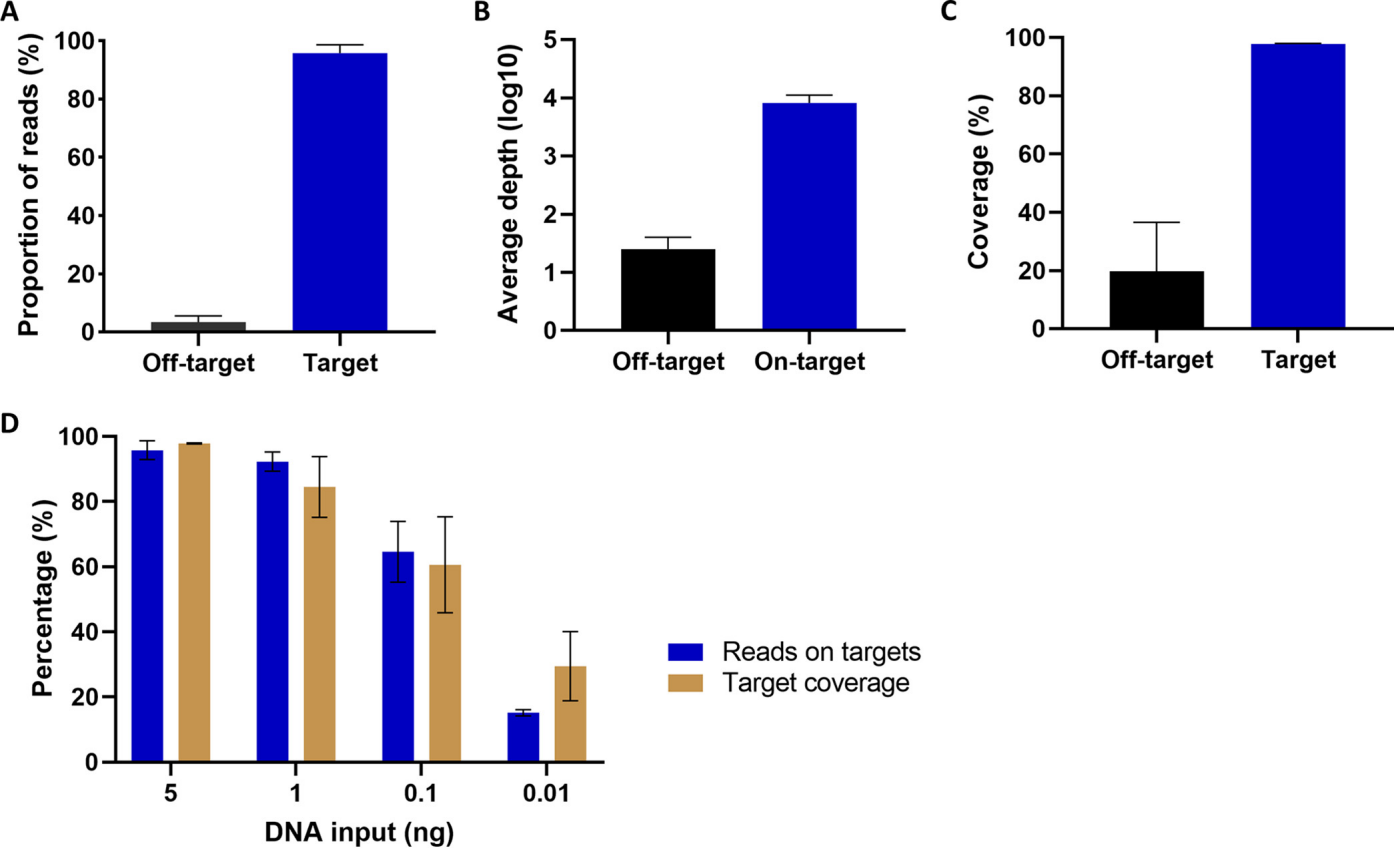
Data quality of FLASH-TB on DNA extract from lab strain H37Rv. (A) Proportion of reads mapped or unmapped to 50 targeted genes. (B) Average depth and (C) Coverage of 50 targeted genes at depth ≥ 10X. (A to C) Five nanogram DNA input. (D) Proportion of reads mapped to 50 targeted genes and their coverage at different amounts of DNA input. The results were presented as mean ± SD from triplicated experiments.

Approximately 8.6 million reads were generated by sequencing cultured H37Rv using FLASH-TB. Of this, 98.0% were mapped to *Mtb* genome and 92.0% mapped to the 50 DR targets (Fig. 1A). As a result, the targeted genes were sequenced at an average depth of 10^4^ (Fig. 1B), whereas for the off-target loci the mean depth was just 32. An average of 97.8% of target sequence was covered with a read depth of at least 10X, compared to 20.0% of the rest of the genome (Fig. 1C). The proportion of on-target reads and coverage dropped with serial dilutions of H37Rv DNA from 5 ng to 0.01 ng (10^6^ − 2 × 10^3^ copies) (Fig. 1D).

**FIG 2:**
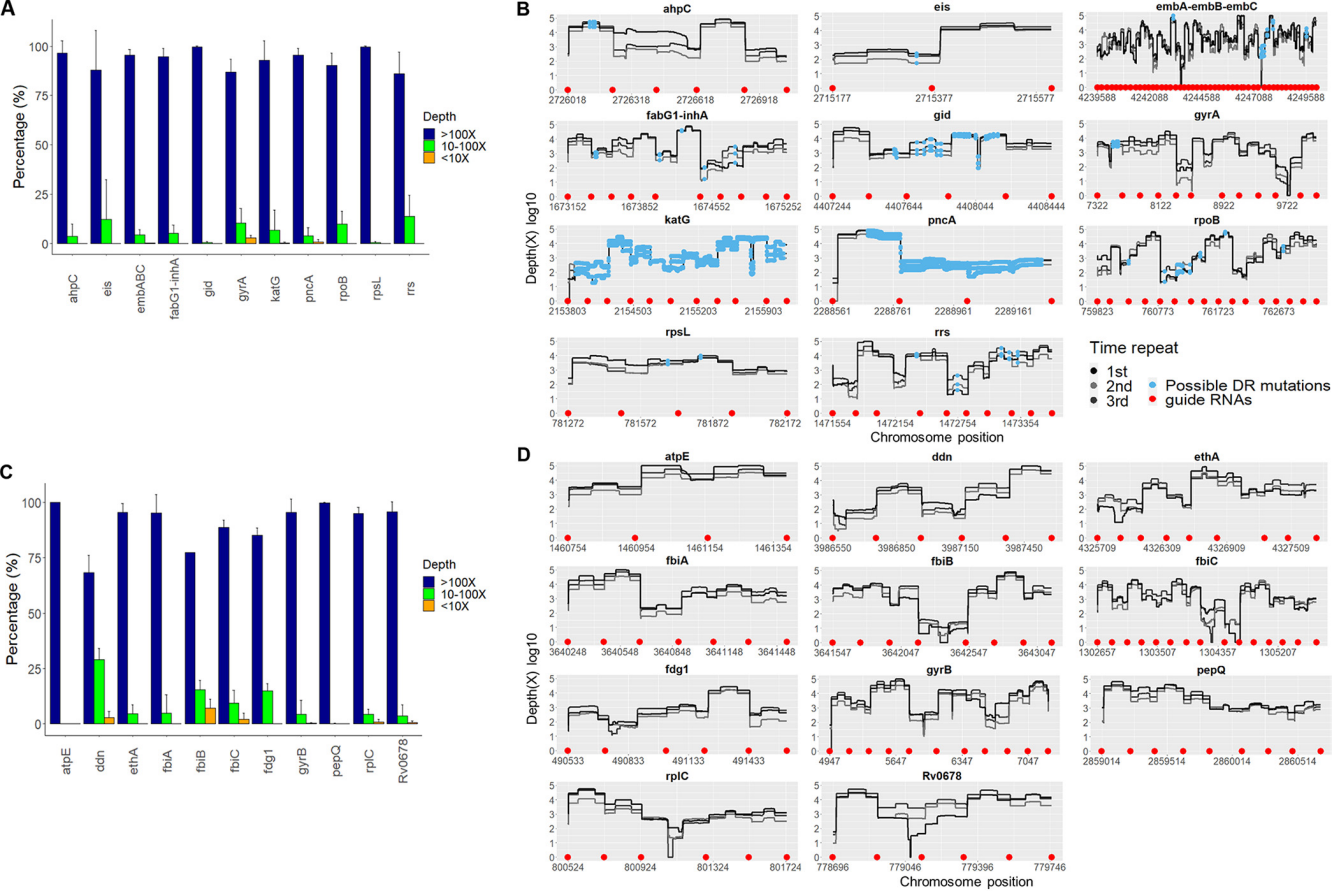
Depth and coverage of individual gene targets included in WHO tier 1 catalogue from FLASH-TB on DNA extract from lab strain H37Rv from independent triplicated experiments. (A and C) Proportion of different depth levels across the individual gene. (B and D) Depth of each single nucleotide across the entire individual gene was plotted. Each line represented for an independent experiment. The blue dots indicated possible mutations associated with drug resistance from Mykrobe catalogue. The red dots indicated the position of guide RNAs where CRISPR/Cas9 targets to cleave. (A and B) Thirteen genes known to be associated with drug resistance from Mykrobe catalogue. (C and D) genes not listed in Mykrobe catalogue, embABC: embA-embB-embC.

We examined those genes sequenced by FLASH-TB that were associated with phenotypic resistance in the WHO catalogue of drug resistance mutations (20). Of these genes, 13 are also featured by Mykrobe (*ahpC*, *eis*, *embB*, *embC*, *inhA*, *fabG1*, *gid*, *gyrA*, *katG*, *pncA*, *rpoB*, *rpsL*, and *rrs*). Almost 100% of *ahpC*, *embB*, *embC*, *inhA*, *fabG1*, *gid*, *pncA*, and *rpsL* had a mean read depth of > 100X, while 75.0% of *eis*, *gyrA*, *katG*, *rpoB*, and *rrs* regions had > 100X coverage, and mean depth for the remaining regions in these genes was between 10 to 100X (Fig. 2A). Two minor regions (approximately 20 bp in length each) in *embB* and *embC* and 2 regions (18 bp, 35 bp in length) in *gyrA* failed to be amplified. However, most positions associated with DR were highly enriched, ranging from 100X to 1000X coverage (Fig. 2B). Of the WHO tier 1 genes not featured in Mykrobe (20), at least 75.0% of *atpE*, *ethA*, *fbiA*, *fdg1*, *gyrB*, *pepQ*, *rplC*, and *Rv0678* had > 100X covered with the remaining of each gene covered by between 10-100X reads (Fig. 2C and D). The exceptions were *ddn*, *fbiB* and *fbiC* for which 5.0% of each gene had a coverage of less than 10X. Some unamplified regions were found in *fbiB* (53 bp), *fbiC* (18 and 73 bp), *rplC* (23 bp), *Rv0678* (2 bp) (Fig. 2D).

### Detection of the DR mutations by FLASH-TB from cultured isolates.

Our 4 clinical isolates carried a total of 17 drug resistance mutations in 7 genes detected by WGS. We next assessed whether FLASH-TB could detect all these mutations and correctly confirm the absence of any DR mutations in the drug-susceptible isolate 5 (Table 1). Mean read depth from FLASH-TB across target genes was again high (approximately 4 × 10^3^) (Fig. S1A), and indeed much higher than from WGS where mean depth ranged between 27 to 40X. Approximately 96.0% of target loci were covered by FLASH-TB at depth ≥ 10X for each strain (Fig. S1B). All 17 DR mutations detected by WGS from culture were also detected by FLASH-TB, but with greater depth for most of them (Table 1). Both WGS from culture and FLASH-TB predicted the same DR profile as pDST for isoniazid, rifampicin, streptomycin, ethambutol, moxifloxacin, and levofloxacin. As no pDST was available for pyrazinamide, no comparison could be made.

**TABLE 1:** Mutations related to drug resistance of *Mtb* clinical isolates detected by cWGS and FLASH-TB

Isolates	cWGS^*a*^			FLASH-TB	
	Drug resistance	Gene-mutation	Depth of coverage of mutation	Gene-mutation	Depth of coverage of mutation
1	MXF/LEV	*gyrA*-D94N	104	*gyrA*-D94N	2076
	EMB	*embA*-M306V	71	*embA*-M306V	345
	INH	*katG*-S315X	61	*katG*-S315X	697
	RIF	*rpoB*-S450X	65	*rpoB*-S450X	114
	STR	*rpsL*-K43R	74	*rpsL*-K43R	1357
2	EMB	*embA*-C-12T	35	*embA*-C-12T	62204
	EMB	*embA*-G406A	27	*embA*-G406A	11727
	INH	*katG*-S315X	44	*katG*-S315X	852
	PZA	*pncA* -117_del_1GG_G	2	*pncA* -117_del_1GG_G	343
	RIF	*rpoB*-H445X	32	*rpoB*-H445X	24
	STR	*rpsL*-K43R	50	*rpsL*-K43R	3879
3	INH	*inhA*-I21T	24	*inhA*-I21T	32048
	INH	*fabG1*-C-15X	45	*fabG1*-C-15X	478
	RIF	*rpoB*-D435X	41	*rpoB*-D435X	27
4	EMB	*embB*-G406S	53	*embB*-G406S	3471
	INH	*katG*-S315X	35	*katG*-S315X	1414
	STR	*rpsL*-K43R	35	*rpsL*-K43R	4543
5	None	None		None	

a cWGS: WGS from culture.

### FLASH-TB for *Mtb* identification and DR prediction from clinical samples.

**FIG 3:**
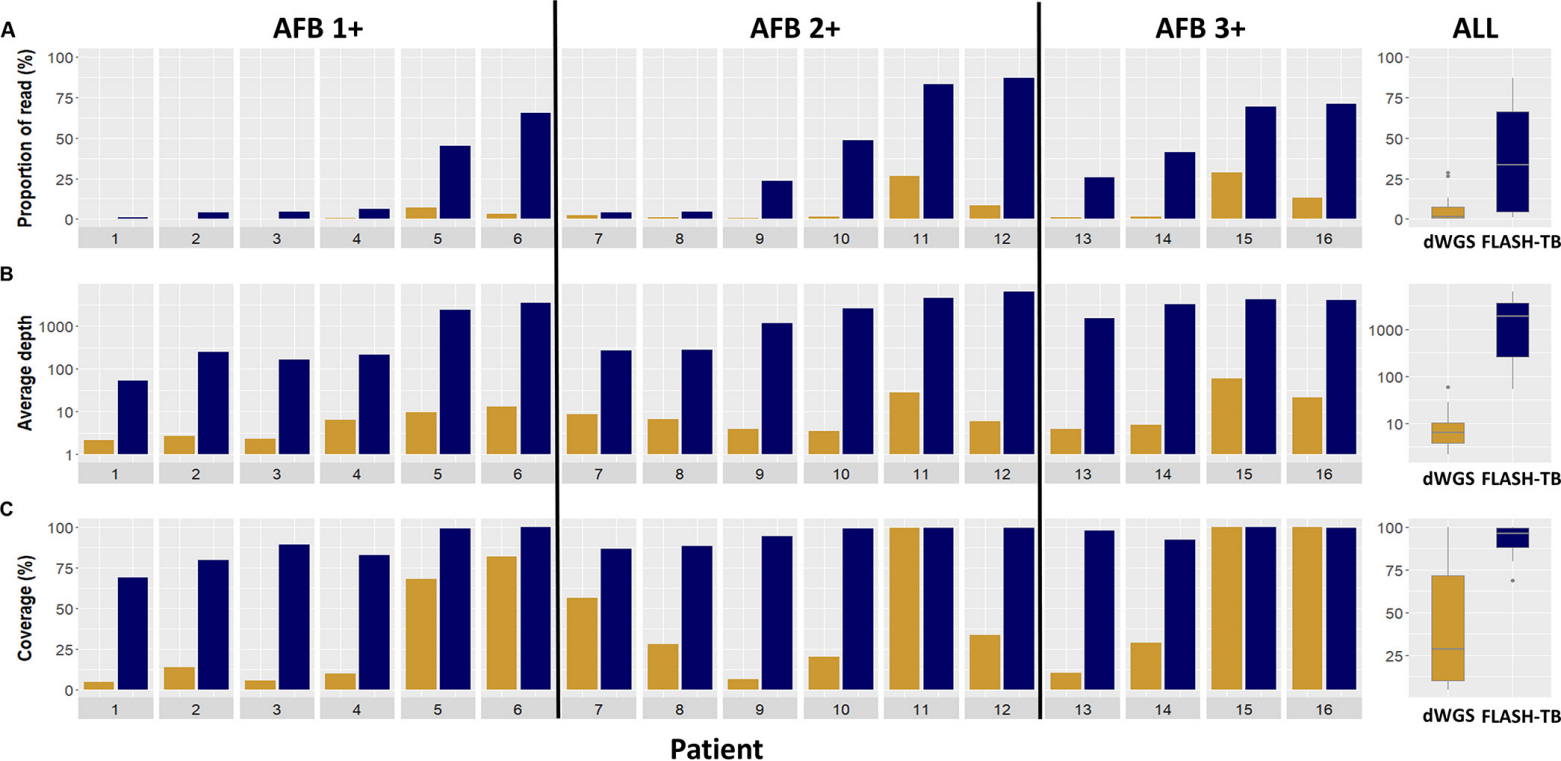
FLASH-TB and dWGS on DNA extracts from sputum of 16 TB patients with different levels of bacterial load determined by Zeihl-Neilsen staining, ranging from AFB (acid-fast bacilli) 1+ to 3+. (A) Proportion of TB reads. (B) Average depth of reads mapped to 50 gene targets. (C) Coverage of 50 targeted genes at depth >3. Gold for dWGS, blue for FLASH-TB from direct sputum.

We next applied FLASH-TB to 16 smear microscopy positive clinical samples. The proportion of TB reads was higher for FLASH-TB than for WGS from direct sputum (dWGS): FLASH-TB median 33.6% (IQR 4.6 to 66.3) versus dWGS median 1.4% (IQR 0.5 to 7.5) (Fig. 3A). A mean 90.1% of TB reads mapped to target genes in FLASH-TB samples, compared to 0.3% of dWGS reads. FLASH-TB produced higher depth of targets compared to dWGS in all 16 samples: FLASH-TB median 1991.0X (IQR 254.4 to 3623.7) versus dWGS median 6.3 (IQR 3.8 to 10.5) (Fig. 3B). FLASH-TB produced a depth of >3 reads for ≥80.0% of target sites for 15/16 (93.7%) samples, whereas dWGS did so for only 4/16 (25.0%) samples (Fig. 3C and Table S5). FLASH-TB yielded ≥ 10X depth for 80.0% of target loci for 13/16 (81.3%) samples, compared to 3/16 (18.8%) for dWGS (Table S5). The genes in the WHO catalogue of drug resistance mutations showed variation in coverage among samples but the majority achieved at least 80% of coverage (median [IQR]): *ahpC* 100 (93.5 to 100), *eis* 100 (86.2 to 100), *embB* 99.3 (91.4 to 100), *embC* 99.2 (95.3 to 100), *inhA*/*fabG1* 99.9 (88.0 to 100), *gid* 97.4 (81.7 to 100), *gyrA* 95.0 (84.4 to 100), *katG* 96.4 (89.6 to 99.5), *pncA* 99.5 (89.7 to 100), *rpoB* 96.7 (87.5 to 100), *rpsL* 100 (98.1 to 100), and *rrs* 99.1 (93.4 to 100). The linear trend test showed the increased bacterial load determined by smear microscopy scores was associated with on-target read depth but not target coverage (Fig. S2). Mykrobe detected *Mtb* complex in 15/16 (93.7%) samples from dWGS data, and determined *Mtb* species in 14/15. FLASH-TB was able to identify *Mtb* complex for 16/16 samples based upon IS*1081* and IS*6110* copies. Target panels of FLASH-TB are not able to identify species within the *Mtb* complex.

Those samples with at least average 80.0% of targets covered with > 3X depth were further analyzed for antibiotic resistance using Mykrobe (Table S5). Predictions were made for 15/16 (93.7%) FLASH-TB samples but only 4 (25.0%) dWGS samples. FLASH-TB detected the same mutations in genes featured in Mykrobe as WGS from culture, with the exception of rpsL (Table 2 and Table S7). In one sample, both sensitive and resistant alleles at rpsL K43R (relevant to streptomycin) were detected by FLASH-TB, but not by culture based WGS (Table 2 and Table S7). Coverage from dWGS direct from this sample was too low to make a call at this site. DR predictions by FLASH-TB were highly concordant with pDST for isoniazid, rifampicin, amikacin, and kanamycin [15/15 (100%)], ethambutol [12/15 (80.0%)] and moxifloxacin [14/15 (93.3%)] (Table S8 and 9). Three patients had ethambutol-resistant genotypes by both FLASH-TB from direct samples and WGS from culture but a sensitive phenotype. Phenotypic resistance to moxifloxacin was observed in one patient although neither WGS from culture nor FLASH-TB directly from sample could detect a relevant mutation (Table S8 and 9). For the 4 samples for which dWGS from sputum made a prediction, these were concordant with predictions for WGS from culture and pDST for all drugs except ethambutol and moxifloxacin (2/4 and 3/4 samples concordance to pDST, respectively) (Table 2 and Table S9).

**TABLE 2:** Concordance of drug resistance detection among FLASH-TB and WGS from direct sputum and culture

	cWGS*^a^* (*n* = 16)			FLASH-TB (*n* = 15)								dWGS (*n* = 4)			
Drug	R^*b*^	r^*c*^	S	R^*b*^	r^*c*^	S	Concordance to cWGS	Case (n)	Control (n)	Sensitivity (95%CI)	Specificity (95%CI)	R^*a*^	r^*b*^	S	Concordance to cWGS
INH^*d*^	4	1	11	4	1	10	15/15 (100%)	5	10	1.0 (0.6-1.0)	1.0 (0.7 to 1.0)	1	1	2	4/4 (100%)
RIF^*e*^	0	1	15	0	1	14	15/15 (100%)	1	15	1.0 (0.2-1.0)	1.0 (0.8 to 1.0)	0	1	3	4/4 (100%)
EMB^*f*^	2	1	13	2	1	12	15/15 (100%)	3	12	1.0 (0.4-1.0)	1.0 (0.8 to 1.0)	1	1	3	4/4 (100%)
KAN^*g*^	0	0	16	0	0	15	15/15 (100%)	0	15	NA	1.0 (0.8 to 1.0)	0	0	4	4/4 (100%)
AMK^*h*^	0	0	16	0	0	15	15/15 (100%)	0	15	NA	1.0 (0.8 to 1.0)	0	0	4	4/4 (100%)
MOX^*i*^	1	0	15	1	0	14	15/15 (100%)	1	15	1.0 (0.2-1.0)	1.0 (0.8 to 1.0)	0	0	4	4/4 (100%)
PZA^*j*^	1	1	14	1	1	13	15/15(100%)	2	13	1.0 (0.3-1.0)	1.0 (0.8 to 1.0)	1	1	2	4/4 (100%)
STR^*k*^	5	0	11	5	1	9	14/15 (93.3%)	6	9	1.0 (0.6-1.0)	0.9 (0.7 to 1.0)	2	0	2	4/4 (100%)

a cWGS, WGS from culture, dWGS: WGS from direct sputum.

b R, resistance was defined when at least 90% of reads of resistance allele were present.

c r, resistance was defined when more than 10% and less than 90% of reads of resistance allele were present.

d INH, isoniazid.

e RIF, rifampicin.

f EMB, ethambutol.

g KAN, kanamycin.

h AMK, amikacin.

i MOX, moxifloxacin.

j PZA, pyrazinamide.

k STR, streptomycin.

## DISCUSSION

Direct sequencing from sputum samples has the potential to detect resistance significantly faster than WGS from MGIT or than culture based pDST. Rapid turnaround times should enable prompt appropriate treatment, benefitting both patients and health services. We described a CRIPSR/Cas9 targeted sequencing method, FLASH-TB, that enriches 52 genes by which to identify *Mtb* complex and predict DR. When applied to a laboratory strain and cultured isolates, FLASH-TB achieved a mean read depth of 1000X and 97.8% target coverage, thus providing large amounts of data on the DR loci covered by Mykrobe. When applied to clinical samples, FLASH-TB detected *Mtb* in all samples and correctly predicted DR in all but one, underlining the potential for this method to rapidly guide patient therapy.

TB treatment guidelines are changing rapidly. For MDR-TB a combination of new and re-purposed drugs is now recommended in a 6-month injection free regimen (21, 22). However, no easy or widely available molecular or phenotypic assay exists for bedaquiline, pretomanid, or linezolid even though WHO aspires to universal DST. The 2021 WHO catalogue of *Mtb* mutations associated with DR included no mutations associated with bedaquiline resistance due to the low prevalence of resistant strains included (20). The wide-spread introduction of new drugs in the absence of standardized DST carries obvious risk of emerging DR, not least because of pre-existing resistance to bedaquiline and lineage 1 specific increases in MICs to pretamanid (23). Molecular diagnostics that can detect resistance early are urgently needed. Deeplex Myc-TB, a commercial targeted next-generation sequencing assay, can detect the resistance mutations for bedaquiline in *Rv0678* and in *rrl* and *rplC* for linezolid, but probes for neither pretomanid nor for delamanid (10). FLASH-TB targets 50 genes previously linked to DR, including to all of the above drugs, and achieves a read coverage of at least 10X across all sites, and > 100X across each of the 3 genes associated with bedaquiline resistance in culture isolates. In sputum samples, the resistance genes, including *rrl*, *rplC*, *Rv0678*, *atpE*, and *pepQ*, had high coverage at depth >3 with a median of 97%, 90.6%, 100%, 100%, and 100%, respectively. As our understanding of the significance of specific mutations across these genes improves, FLASH-TB could provide a comprehensive DR profile directly from sputum samples in the future.

Extracting DNA from sputum remains challenging, with low DNA yields and high levels of contaminating DNA from human host cells and other bacteria commonly found in sputum (9, 24). Whereas insufficient coverage was obtained for most targets via dWGS from sputum, FLASH-TB performed markedly better, not only achieving good coverage but also producing results that were concordant with WGS from culture and pDST. For 3 patients, ethambutol-resistance was predicted genotypically by FLASH-TB but pDST indicated susceptibility. This discordance likely resulted from peri-threshold MICs conferred by 2 mutations, embB_G406D or embB_M306I (25). One *Mtb* strain was predicted susceptible to fluoroquinolones by FLASH-TB but resistance by pDST, suggesting the presence of a resistance mechanism (26). FLASH-TB has a number of other advantages. Its design is flexible such that crRNAs could be introduced or removed from the pool of crRNAs as the list of purported DR genes changes. FLASH-TB can deliver a test result within 48 h for a local estimated consumable cost of 275 USD per sample, which is similar to WGS from direct sputum. However, due to the low uptake of sequencing, the local cost of FLASH-TB is currently higher than pDST for both first- and second-line drugs (75 USD) alone or combined with line-probe assays to deteremine species and resistance (150 USD).

This study has several limitations. For H37Rv, FLASH-TB generated less than 10X coverage for a few small regions in genes such as *ddn*, *fbiB*, and *fbiC*. One possibility is that the guide RNAs for these regions failed although we cannot be entirely sure. As data on *in vitro* failure patterns improve in future, the reasons will become more apparent (13). As no official DR mutation catalogues exist for these genes anyway, the impact of low coverage in these regions remains limited. FLASH-TB shares some limitations with other targeted sequencing approaches, particularly with regard to the detection of deletions, where it is hard to distinguish these from failed amplification. Moreover, Mykrobe has also not been tuned to detect indels larger than 10 nucleotides. In this study, FLASH-TB was performed only on a limited number of clinical samples, all of which were positive on smear microscopy. Few samples were resistant to rifampicin or isoniazid as these were collected from a nonselective observational study of TB in Ho Chi Minh city where for MDR-TB incidence is about 4.1% (27). Some regions of genes associated with DR in the 2021 WHO catalogue were not covered in several of our clinical samples, although these were almost fully covered in H37Rv. It is likely that the low number of bacteria in these samples was to blame, as we demonstrated that target coverage drops with the amount of H37Rv DNA. Further work is, therefore, required to evaluate samples with very low *Mtb* load including scanty smear positive and smear negative samples, as well as larger collections containing more DR. The performance of FLASH-TB compared with commercial targeted Deeplex Myc-TB or other targeted WGS approaches also needs to be evaluated.

We have demonstrated that FLASH-TB successfully amplifies target genes which are then sequenced to high coverage and depth from both laboratory strains and clinical cultures. This technique has high multiplexing capacity, with flexibility around target selection as global databases of resistance-associated mutations update. FLASH-TB could therefore prove a useful technique for detecting *Mtb* DR directly from sputum samples.
