# Supplementary material for: FLASH-TB: an Application of Next-Generation CRISPR to Detect Drug Resistant Tuberculosis from Direct Sputum
Source: J Clin Microbiol. 2023 Apr 3;61(4):e01634-22. doi: 10.1128/jcm.01634-22 (PMC10117099; doi:10.1128/jcm.01634-22)
Supplement: Supplemental file 1 — Supplemental material. Download jcm.01634-22-s0001.pdf, PDF file, 0.2 MB [file jcm.01634-22-s0001.pdf]

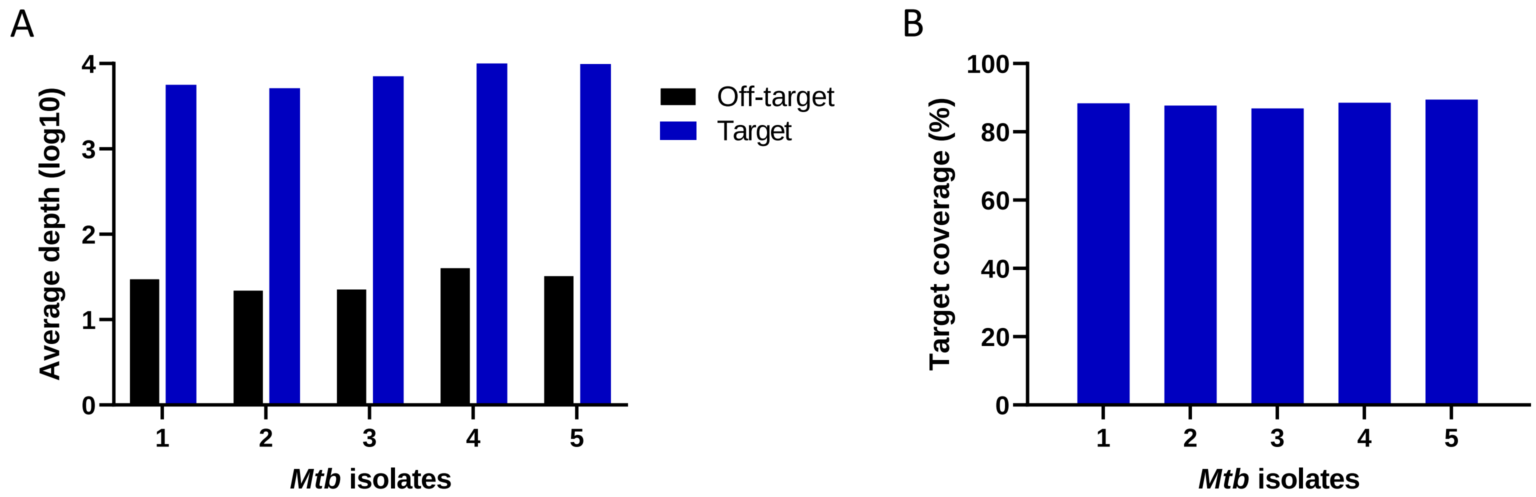

Supplementary Figure 1. Data quality of FLASH-TB with DNA of *Mtb* clinical isolates. (A) Average depth (log10) of targets or off-targets. (B) Target coverage of 52 genes.

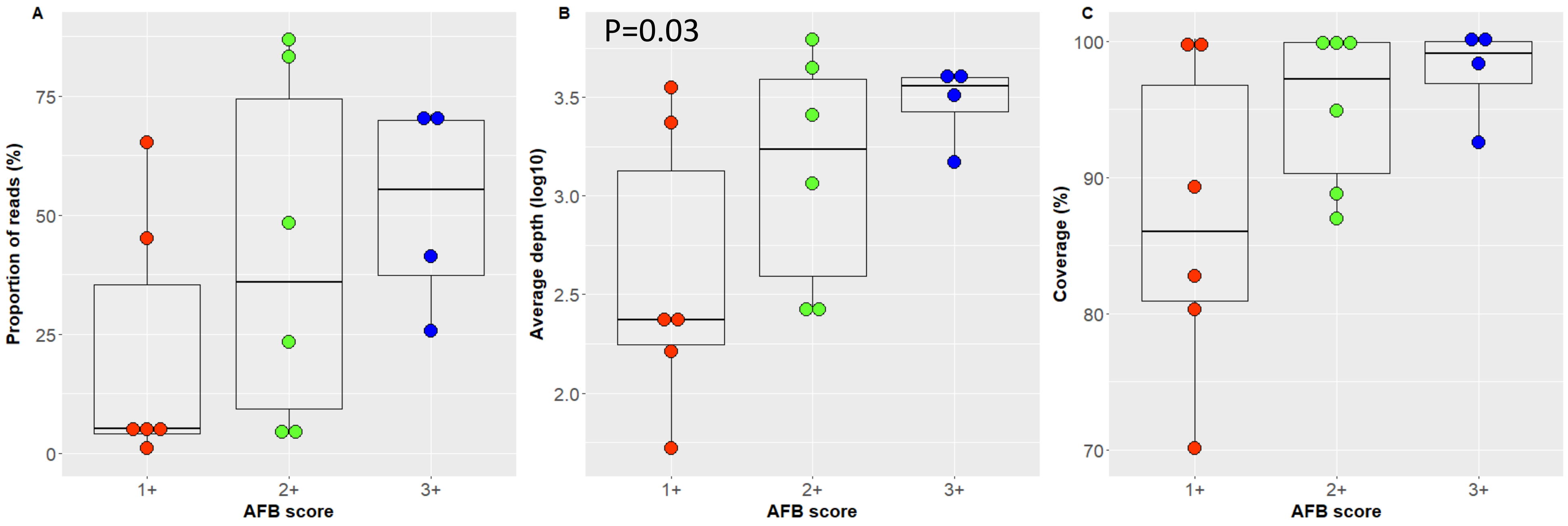

Supplementary Figure 2. Correlation between AFB scores and data quality for FLASH-TB from 16 sputum samples. (A) AFB score and proportion of TB reads . (B) AFB score and average depth of gene targets. (C) AFB score and target coverage at depth >3. P: P-value of trend test.
